# Digital health technologies for accessing contraceptive services among young people in Sub-Saharan Africa: A scoping review protocol

**DOI:** 10.1371/journal.pdig.0000748

**Published:** 2025-07-10

**Authors:** Agnes Kyamulabi, Eva Oberle, Lotenna Olisaeloka, Innocent Kamya, Ingrid Nyesigire, Wendy V. Norman, Abdul-Fatawu Abdulai

**Affiliations:** 1 School of Population and Public Health, University of British Columbia, Vancouver, Canada; 2 School of Social Sciences, Makerere University, Kampala, Uganda; 3 Department of Family and Practice, University of British Columbia, Vancouver, Canada; 4 School of Nursing, University of British Columbia, Vancouver, Canada; Iran University of Medical Sciences, IRANISLAMIC REPUBLIC OF

## Abstract

This scoping review aims to examine and synthesize existing literature on the use of digital health technologies, with a focus on the extent and types of technologies used to access contraceptive services among young people in Sub-Saharan Africa (SSA). Globally, digital health technologies have emerged as pivotal tools in addressing contraceptive needs among young people. In SSA, where traditional healthcare systems often face numerous challenges, these technologies offer innovative solutions to improve access to contraceptive services. Despite growing interest in digital health technologies, comprehensive reviews on contraceptive access among young people in SSA are still lacking. Most existing studies focus broadly on sexual and reproductive health (SRH) or adult populations, leaving a gap in understanding the unique needs and experiences of young people using digital technologies for contraception services. It is unclear how much research has been conducted to examine how these technologies can facilitate contraceptive use, which technologies are used and why, where this evidence is concentrated within SSA, and the prevailing gaps. Therefore, we propose to undertake a scoping review. This scoping review will include studies focusing on young people aged 10–24 years in SSA, addressing access challenges to contraceptive services within this age group. The review will consider client-facing digital health technologies. All methodological approaches and designs will be included. Reviews, protocols, conference papers, policy briefs and studies conducted outside SSA will be excluded. The review will apply the comprehensive search strategy recommended by Joanna Briggs Institute (JBI). The initial limited search of MEDLINE (Ovid) and CINAHL Complete (EBSCOhost) was conducted with guidance from the University Librarian. This informed the selection of keywords, along with index terms, to develop a full search strategy for MEDLINE (Ovid), CINAHL Complete (EBSCOhost), Scopus, Compendix Engineering Village, and IEEE Xplore. The scoping review shall also use supplementary resources such as google scholar, and African Journal online (AJOL). We will also review the reference lists of articles that meet the inclusion criteria to ascertain articles that were not returned by the search criteria. Data will be presented using tables and charts, accompanied by a narrative summary. This scoping review was registered in Open Science Framework: **https://doi.org/10.17605/OSF.IO/5QJ6P.**

## Author summary

Young people in Sub-Saharan Africa continue to face major challenges in accessing contraception, including stigma, lack of information, and limited youth-friendly services. Digital health technologies such as mobile apps, SMS-based or text messaging, and web-based or online platforms have the potential to reduce these barriers by providing private, accessible, and youth-centred support. However, it remains unclear what technologies are being used across the region, how widely they are adopted, and what gaps still exist in reaching young people. In this study, we outline a protocol for a scoping review that will systematically map the evidence on digital health technologies used to access contraceptive services among young people aged 10–24 years in Sub-Saharan Africa. Our review will identify the types of technologies being used, their geographic distribution, and the key opportunities and challenges they present. By providing a clear picture of what is currently known and where further research is needed, our work aims to inform future studies, policy efforts, and digital interventions that support young people’s reproductive health across the region.

## Introduction

Access to contraceptive services for young people is essential for their physical health, social empowerment, and economic well-being, as it facilitates informed decision-making and prevents unintended pregnancies. The term “young people” refers to adolescents (10–19 years) and youths (15–24 years), as defined by the United Nations (UN) [1,2]. Globally, young people constitute about 1.8 billion of the population [3,4], emphasizing the need for contraceptive initiatives to prepare them for adult life before engaging into child birth. This is because adolescent pregnancy and childbirth come with several complications which are the leading cause of death among girls aged 15–19 years globally [5]. In 2022, an estimated 13% of adolescent girls and young women globally gave birth before the age of 18 [6]. Guttmacher Institute reported that each year, around 21 million girls aged 15–19 in developing regions become pregnant, with approximately 12 million giving birth [7]. In 2023, the global adolescent birth rate for women aged 15–19 stood at 41.3 births per 1,000 women, while that for girls aged 10–14 was 1.5 per 1,000. [8] Notably, 10% of all births in 2023 were to adolescent mothers [8]. Despite a general increase in use of modern contraceptives [8], the proportion of adolescents and young women whose contraceptive needs are met remains lower than that of older women [7,9]. This highlights ongoing challenges in ensuring equitable access to contraceptive services for younger populations

Adolescent pregnancy significantly contributes to maternal and child mortality [5] and perpetuates the cycle of ill-health and poverty. Despite the numerous risk factors associated with adolescent pregnancies, contraception knowledge and use of modern contraception remain low in many developing nations [9]. The global agenda set by the United Nations’ Sustainable Development Goals (SDGs) for 2030, particularly Goals 1, 3, 4, and 5, emphasizes the importance of enhancing health and promoting gender equality [8,10]. These health-related goals highlight the critical need for equitable access to contraception. Goal 3.7 focuses on achieving universal access to sexual and reproductive healthcare services, including family planning information and education, while integrating reproductive health into national policies by 2030. Regardless of these ambitious plans, a substantial number of adolescents and young women worldwide continue to experience an unmet need for contraception [7]. Unmet need here describes women who do not use modern contraception despite wanting to delay pregnancy or do not want more children [7,11]. Addressing the unmet need for modern contraception among women in low-income regions could significantly reduce the number of unintended pregnancies and unplanned births by 68% annually [7,12].

Contraceptive use among young people in Sub-Saharan Africa (SSA) is a significant concern due to unique demographic and health challenges. Young people constitute a third of the region’s population [13], and SSA bears a considerable burden of unintended pregnancies, particularly among adolescents [12,14,15]. High rates of adolescent pregnancies, and unmet needs for contraception are prevalent [14]. In 2023, Sub-Saharan Africa recorded the highest adolescent birth rates in the world, with 97.9 births per 1,000 women aged 15–19 and 4.4 per 1,000 girls aged 10–14 [5,8,9,16]. In spite of a global decline in adolescent birth rates—dropping from 64.5 per 1,000 in 2000 to 41.3 in 2023—the region continues to have a disproportionately large number of adolescent pregnancies, with over 6.1 million births to women aged 15–19 occurring in Sub-Saharan Africa [9,16]. Adolescent pregnancies not only disrupt girls’ development but also create lasting barriers to their education, employment prospects, and overall health [17].

### Digital health for contraception services

In SSA, 68% of adolescents have unmet contraceptive needs [7]. Barriers to contraception include cost, stigma, concerns over potential side effects, lack of knowledge, legal, political, structural barriers, and socio-cultural norms [7,9,12,18–23]. Given the challenges in contraceptive access, digital health is seen as an alternative or complementary mode of providing contraception services to young people. In this review, digital health includes the application of information and communication technology to aid health and health-related areas [24]. The COVID-19 pandemic accelerated the adoption of technology and hastened the transition to digital tools [25,26]. Technology-based and technology-driven interventions have emerged as tools for empowering young people [25], as they can play a crucial role in overcoming some of the barriers to contraceptive uptake among young people in Sub-Saharan Africa, both directly and indirectly. Given the high burden of unintended pregnancies among young people in Sub-Saharan Africa (SSA) and the transformative potential of digital health technologies, it is crucial to focus on specific interventions that can yield significant benefits, and this is where specifically young people’s access to the technology is important. Digital health technologies, such as mobile health (mHealth) applications, telemedicine, telehealth, e-health, social media and web-based educational resources, offer accessible, confidential, and user-friendly contraception and information services [27]. A qualitative study conducted in higher-income countries revealed that telemedicine for abortion services has the potential to reduce access barriers that were already present before the pandemic [26]. Understanding the most commonly used technologies in contraception service delivery among young people in Africa and their preferences can optimize their deployment [28] and in the long run, they can have a contribution toward reducing unintended pregnancies among young people. For example, mHealth applications have been used to provide contraceptive education, reminders, and connections with healthcare providers [11,29,30].

Although there is growing interest in digital health technologies, comprehensive reviews examining the types of digital health technologies used, their concentration in Sub-Saharan Africa (SSA), and barriers and facilitators to use are still lacking. Most existing studies focus broadly on sexual and reproductive health (SRH) or adult populations, creating a gap in understanding the unique needs and experiences of young people in SSA who may rely on digital tools for contraception services. For instance, Ninsiima et al. [31] examined factors influencing access to youth-friendly SRH services in Africa; Obiezu-Umeh et al. [32] assessed the effectiveness of implementation strategies for youth-friendly SRH in SSA; and Feroz et al. identified a variety of mHealth solutions aimed at enhancing young people’s sexual and reproductive health (SRH) in low- and middle-income countries (LMICs) [33]. These studies contribute to a broad understanding of SRH but offer limited insights into how digital technologies specifically contribute to contraceptive access for young people in SSA. Therefore, questions remain on how these technologies facilitate contraceptive use, the types of digital tools used, where the evidence is concentrated, barriers and facilitators and where gaps in knowledge exist. A scoping review is well-suited to address these questions by systematically examining the breadth and nature of evidence on this topic and identifying gaps.

An initial search of Epistemonikos, the Cochrane Database of Systematic Reviews, and MEDLINE confirmed that no reviews on digital contraceptive services for young people in SSA have been registered or conducted. Some systematic reviews have assessed digital interventions aimed at improving contraceptive use, such as Perinpanathan et al.‘s review of mobile-based interventions [11], and Inhae & Jiwon’s meta-analysis of mHealth interventions’ effects on contraceptive use and pregnancy occurrence among young people, focusing only on randomized control trials [34]. However, no review specifically maps the digital health technologies used to facilitate contraceptive services among young people in SSA. Context differences make reviews from high-income countries less applicable, while reviews focusing on LMICs often overlook SSA nations, limiting their relevance. Thus, this scoping review aims to understand the extent and type of digital health technologies used to access contraceptive services among young people in SSA. The review will synthesize evidence on digital technologies facilitating contraceptive services among SSA’s young people, identifying their geographic concentration, exploring associated challenges and opportunities, and highlighting future research needs. Ultimately, findings will inform future research on contraceptive use and unintended pregnancies in SSA’s young population and eventually contribute to improving contraception outcomes. The detailed recommendations, including those addressing large-scale implementation and feasibility or technical barriers, will be developed and fully captured in the final scoping review which is currently underway.

## Methods

The proposed review will adopt the Joanna Briggs Institute (JBI) methodology for scoping reviews [35]. It will adhere to the PRISMA-ScR (Preferred Reporting Items for Systematic reviews and Meta-Analyses extension for Scoping Reviews) guidelines for reporting [36], (S1 Appendix) ensuring a comprehensive and transparent approach to mapping the evidence. These guidelines will help to ensure that all key elements of scoping reviews are accurately and clearly presented. This scoping review will identify and map out the various technologies used to access contraceptive services among young people. The final review report will provide practical guidance and policy-relevant recommendations drawn from the mapped evidence.

## Review questions

1. What digital health technologies are currently used to access contraceptive services among young people in Sub-Saharan Africa?
2. Which Sub-Saharan African countries is this evidence concentrated?
3. What are the key opportunities and challenges associated with using digital health technologies to access contraceptive services among young people in Sub-Saharan Africa?
4. What recommendations can be drawn from existing literature to guide future research in leveraging digital health interventions to improve contraception outcomes among young people in Sub-Saharan Africa region?

### Search strategy

This scoping review will apply the comprehensive three-stage search strategy recommended by JBI [35] to locate both published and unpublished studies. First an initial limited search of MEDLINE (Ovid) and CINAHL Complete (EBSCO) was undertaken to identify articles on the topic. This was done with guidance from University Librarian. Second, the text words contained in the titles and abstracts of relevant articles and the index terms used to describe the articles were used to develop a full search strategy for the different databases to be searched (S2 Appendix). The search strategy, including all identified keywords and index terms, will be adapted for each included database and/or information source. Third, the reference list of identified or included reports and articles will be searched for additional sources. This will specifically focus on examining the reference lists of the sources that will be selected from full-text and or included in the review.

The following databases will be searched: MEDLINE (Ovid), CINAHL Complete (EBSCOhost), Scopus, Compendix Engineering Village, and IEEE Xplore. These databases cover a wide range of disciplines, including health sciences and technology. We shall retrieve the search results and export them into Covidence software tool [37] as guided by the librarian to facilitate organization and removal of duplicates. The scoping review shall also use supplementary resources such as google scholar, and African Journal online (AJOL). For Google Scholar, the advanced search feature will be applied, where we will input the same keywords that were used in the Medline (Ovid) search. The search results will then be bulk exported using the Publish or Perish software for further analysis. For African Journal Online (AJOL), we will manually search the platform using the same set of keywords and inclusion criteria to ensure consistency. Relevant studies identified will be individually exported for inclusion in the review. Due to limited resources, only English-language articles and research reports will be considered for inclusion. To avoid bias relating to publication date and to capture the full spectrum of available literature, no date restrictions will be applied. Although some exclusion criteria were not directly applied as filters during the database search, all identified papers will undergo a rigorous screening process. The predefined criteria will be systematically applied at all the screening stages, in accordance with the Joanna Briggs Institute (JBI) guidelines for scoping reviews [35,38]. This will ensure a comprehensive yet focused selection of studies relevant to the review objectives.

### Eligibility criteria

#### Population.

The scoping review will include papers on digital health interventions providing contraceptive services to young people (aged 10–24 years) in Sub-Saharan Africa (Table 1). This review adopts the UN definition of young people which encompasses adolescents (aged 10–19) and youths (aged 15–24). The African Union considers a youth or young person to be between the ages of 15–35 years [13,39]. Although this definition represents regional political and socioeconomic realities [13], this review will utilize the global definition because it captures the important transitional period from childhood to adulthood [1]. This review will include studies focusing on the general population of young people, without specific reference to marginalized or high-risk subgroups (e.g., refugees, sexual minorities, economically disadvantaged youth). Any study that primarily focuses on women of reproductive age (15 – 49) will be excluded.

**Table 1:** Summary of inclusion and exclusion criteria for paper selection.

Criteria	Inclusion	Exclusion
Population	Studies primarily focused on young people (aged 10–24 years)	Studies not focused on this age group
Concept	Studies on digital health technologies for accessing contraceptive services, particularly client facing digital tools	Studies not addressing access to contraception using digital health, including papers on the broader SRH spectrum, such as STI and HIV/AIDS care
Context	Studies conducted in Sub-Saharan Africa	Studies conducted outside of SSA
Language	Papers written in English	Papers not written in English language
Timeframe	No limit on timeframe to capture all relevant publications in the databases searched.	None based on timeframe
Study Types	Empirical research articles (quantitative, qualitative, mixed methods); research/evaluation reports, dissertations, & theses.	Reviews, protocols, commentaries, policy briefs, opinion papers, conference abstracts

#### Concept.

“Digital health technologies” is the main concept to be explored in this review. Digital health originates from the concept of eHealth, which refers to the application of information and communication technology to aid health and health-related areas [24]. The review will consider studies with a focus on client facing digital health technologies that provide contraceptive services including contraception information, scheduling appointments, consultations, menstruation tracking and links to healthcare services. The review will cover various digital health technologies such as mobile apps, video consultation, telephone calls, text messaging, social media campaigns, and web-based health educational resources. Contraception services are the primary outcome due to the significant access challenges faced by this age group and opportunities presented. Studies addressing contraception outcomes like access, delivery, menstrual health, contraceptive information, family planning counselling, and related topics will also be included. Articles that focus exclusively on service provider-oriented tools, such as electronic health records, digital prescription systems, and billing or insurance platforms, will be excluded. Additionally, studies that primarily address the physical provision of contraceptive services, such as IUD insertion, will not be included. Furthermore, any papers that do not specifically address the use of digital tools for contraceptives or pregnancy prevention but instead focus on other aspects of the broader sexual and reproductive health spectrum, such as STI and HIV/AIDS care, will also be excluded.

#### Context.

Studies conducted in Sub-Saharan Africa (SSA) will be considered and eligible for inclusion if they meet all the other inclusion criteria. World Bank defines SSA as countries that lie south of the Sahara, and these are forty-eight countries [40]. These same countries also make up the World Health Organization African Region [41]. Twenty-four of the twenty-seven countries in the World Bank’s Low-income classification are in SSA. The remaining SSA countries are mostly Lower-Middle income. SSA has the highest youth poverty rate in the world (70%) [42], a factor contributing to the high regional prevalence of teenage or adolescent pregnancy. The review will consider interventions implemented in settings such as schools, universities, health centres and communities within sub-Saharan African countries. Given the context of the subject, all studies conducted within a school or university setting will be included. Studies conducted outside SSA or studies that have a combination of countries including those from SSA and those from different continents shall be excluded.

### Types of sources

This scoping review will consider any type of source, without limitations on the methodological approach, including quantitative, qualitative, and mixed-methods study designs. The sources of information will include scholarly journal articles. Research or evaluation reports, dissertations and theses are the only grey literature that will be included in the review. However, reviews, protocols, commentaries, policy briefs, conference papers, and opinion papers will not be included in this review. By excluding these, we aim at including only primary, peer-reviewed empirical studies with original data relevant to the review objectives. The excluded sources often lack detailed methodology or do not contribute new findings suitable for mapping the landscape of digital contraceptive initiatives. In particular, the reviews were excluded from data extraction to avoid duplication of primary study data.

### Study selection

Following the search, all identified citations will be uploaded into Covidence software [37]. Duplicates will automatically be removed. A pilot test will be carried out by having two independent reviewers screen at least 5% of the titles and abstracts based on the inclusion criteria. The results will then be discussed, and any necessary adjustments to the inclusion and exclusion criteria will be made in collaboration with the team. After completing the pilot test, two independent reviewers will screen the titles and abstracts of all eligible sources using the inclusion and exclusion criteria. The full text of selected citations will be assessed in detail by two independent reviewers and those that will not meet the inclusion criteria will be excluded and reasons for excluding them will be clearly documented in the final review report. Any disagreement that arises between reviewers at each stage of the selection process will be resolved through a discussion. The results of the search and the study inclusion process will be reported in full in the final scoping review and presented in a Preferred Reporting Items for Systematic Reviews and Meta-Analyses extension for Scoping Review (PRISMA-ScR) flow diagram [36]. In accordance with scoping review guidelines, we will not assess the methodological quality or risk of bias of the studies included in our review [36,43].

### Data extraction

Data extraction process will be guided by Joanna Briggs Institute (JBI) recommendations for data extraction [44]. Data will be extracted by two independent reviewers using a JBI modified data extraction tool [38] (S3 Appendix). This will also be piloted by 2 independent reviewers using at least 2 papers per source type to ensure consistency; and may be iteratively modified as the review progresses. In situations where additional relevant data items are identified, reviewers will discuss and agree on the items added on the extraction tool and eventually report about the deviation from the protocol and provide the justification [44]. During pilot-testing of the extraction tool the reviewers will reflect on whether there is anything missed on the extraction tool, anything redundant in the extraction tool, whether there is anything in the extraction tool that calls for further clarification, and the time taken to extract the necessary information [44]. All this information will help to guide further the extraction process. The data extracted will include specific details about population characteristics such as country, sample size, age and sex/gender; types of digital health technologies used; contraceptive services accessed using those technologies; study methods and key findings relevant to the review question/s. We shall also extract the title of the papers, authors, objective, evidence concentration, intervention characteristics, key outcomes, gaps and opportunities. Any disagreement that arises between the reviewers at each stage of data extraction process will be resolved through a discussion. If appropriate, authors of the included papers will be contacted to request missing or additional data, where required.

### Stakeholder involvement

Stakeholder involvement or consultation is a key aspect of scoping reviews [45]. Levac and colleagues authors argue that “consultation should be considered a required component of scoping study methodology, rather than optional step” [45]. Consulting stakeholders would help to enhance the relevance, contextual accuracy, and utility of the findings. However, while this review does not include a formal stakeholder involvement phase, the composition of the research team itself reflects meaningful stakeholder representation. The co-authors include clinicians, adolescent health experts, psychologists, digital health experts, and users of digital contraceptive tools, several of whom are based in or have significant experience working in Sub-Saharan Africa. This multidisciplinary and contextually grounded team was intentionally assembled to ensure that the synthesis reflects lived experience, professional expertise, and regional insight relevant to the review topic.

### Data analysis and presentation

For the mapping process, we will create a detailed descriptive map to visually represent key characteristics and the distribution of the studies included in the review. This map will categorize studies based on variables such as population characteristics, geographic location, study design, methodologies used, and key outcomes. To systematically capture this information, we will use a matrix that will allow for the identification of trends, gaps, and the overall scope of the existing literature. This approach aims to point out areas where evidence is concentrated and highlight gaps, thus providing a comprehensive view of the research landscape. We hope to analyse and synthesize the data using basic descriptive statistics such as percentages or proportions; and also apply basic qualitative content analysis, in keeping with the approach to scoping reviews analysis by Pollock *et al.* [44]. Two independent reviewers will conduct the basic qualitative content analysis and it will be formed through categorization of the types of technologies; whether the evidence focuses on facilitating access to contraceptive services, challenges and opportunities, and any differences in contraception outcomes; if at all it exists.

A narrative summary will accompany these visual presentations, describing the relationship between the review questions and the findings. Initially, the presentation will detail the authors, publication years, types of sources, settings, study designs, geographic locations, delivery formats, and populations involved in the studies. The second set of data presentation will focus on the digital technologies and applications used to access contraceptive services among young people in Sub-Saharan Africa. It will highlight opportunities the technologies present, discuss challenges faced, and identify gaps in the current research. The presentation will conclude with recommendations for future research and implications for policy and practice.

### Conceptual frameworks for synthesis and interpretation

We will draw upon two conceptual frameworks during the analysis, synthesis and discussion to situate the interpretation of the results and to contextualise the utilisation of digital tools for contraceptive access among young people SSA. The Social Determinants of Health (SDoH) framework will be applied to situate digital tools within broader social and structural contexts, including gender, education, income, digital access, and youth-friendly service availability [46]. This lens will support analysis of how social inequalities shape both access to and utilization of digital contraceptive initiatives. We will also apply the Unified Theory of Acceptance and Use of Technology (UTAUT) to assist with interpreting how young people engage with digital technologies for contraceptive use [47,48]. Constructs such as “performance expectancy, effort expectancy, social influence, and facilitating conditions” will be unpacked to explore facilitators and barriers to adoption of mHealth initiatives. Together, these frameworks will guide the interpretation of findings in the discussion section, allowing us to map evidence across social, behavioural, and technological domains while remaining grounded in our review’s core objectives.

## Supporting information

S1 Appendix PRISMA checklist. (PDF)

S2 Appendix Search strategy. (PDF)

S3 Appendix Data extraction tool. (PDF)
