# Supplementary material for: Digital health technologies for accessing contraceptive services among young people in Sub-Saharan Africa: A scoping review protocol
Source: PLOS Digit Health. 2025 Jul 10;4(7):e0000748. doi: 10.1371/journal.pdig.0000748 (PMC12244615; doi:10.1371/journal.pdig.0000748)
Supplement: S2 Appendix — (PDF) [file pdig.0000748.s002.pdf]

S2 Appendix: Search strategy

Ovid MEDLINE(R) ALL <1946 to July 27, 2024>

Sequence	Search Terms	Hits
**1.**	exp Adolescent/	2261543
2.	adult children/ or young adult/ or adolescent parent/ or young person/ or young people/	1050879
3.	(Young people or Young person* or youth* or adolescen* or young adult* or young* or teen* or student* or Adult Child* or Adolescent Parent* or Adolescent Mother* or Adolescent Father* or high school* or secondary school* or college or university).mp. [mp=title, book title, abstract, original title, name of substance word, subject heading word, floating sub-heading word, keyword heading word, organism supplementary concept word, protocol supplementary concept word, rare disease supplementary concept word, unique identifier, synonyms, population supplementary concept word, anatomy supplementary concept word]	4159974
4.	exp Adolescent Health Services/ or Adolescent Health/	7907
5.	1 or 2 or 3 or 4	4159974
**6.**	exp Digital Health/ or exp Internet/ or exp Mobile Applications/ or exp Telemedicine/	154857
**7.**	(Digital Health* or Mobile Health* or mhealth or mobile app* or telemedicine or telehealth or ehealth or e-health or emedicine or digital technolog* or digital medicine or online health* or virtual health or online education* or social media or instagram or facebook or whatsapp or text messag* or SMS or online forum or online health education).mp. [mp=title, book title, abstract, original title, name of substance word, subject heading word, floating sub-heading word, keyword heading word, organism supplementary concept word, protocol supplementary concept word, rare disease supplementary concept word, unique identifier, synonyms, population supplementary concept word, anatomy supplementary concept word]	162744
**8.**	6 or 7	242579
**9.**	exp Sexual Health/ or exp Reproductive Health Services/ or exp Reproductive Health/	52049
**10.**	exp Abortion, Induced/	43692
11.	exp Family Planning Services/	26847

12.	exp Reproductive Medicine/	25021
**13.**	(sexual health or reproductive health or SRH or reproductive medicine or reproductive services or family planning or family planning servic* or health servic* or health promotion or sexual health services or postnatal care or pre-pregnancy care or prenatal care or maternal health servic* or birth interv* or sexual health clini* or abortion* or contraceptive* or contraception or emergency contracept* or birth control or contraceptive pill* or contraceptive implant or morning after pill or cocp or emergency pill or intrauterine device* or condom* or wearable devic* or chatbot* or clue or flo or besid* or barrier method* or abstinence or menstruation or menstrual track* or period track* or teen* pregnancy or adolescent pregnancy or antenatal or fertility or sex education or sexual health education or sexuality).mp. [mp=title, book title, abstract, original title, name of substance word, subject heading word, floating sub-heading word, keyword heading word, organism supplementary concept word, protocol supplementary concept word, rare disease supplementary concept word, unique identifier, synonyms, population supplementary concept word, anatomy supplementary concept word]	1308215
**14.**	9 or 10 or 11 or 12 or 13	1327453
**15.**	exp South Africa/ or exp "Africa South of the Sahara"/ or exp Africa, Central/ or exp Africa, Northern/ or exp Africa, Southern/ or exp Africa, Eastern/ or exp Africa/ or exp Africa, Western/	339919
**16.**	(((Sub-Saharan Africa or Africa* or West* Africa or East* Africa or South* Africa or Central Africa or Angola or Benin or Botswana or Burkina Faso or Burundi or Cape Verde or Cameroon or Central African Republic or Chad or Comoros or Democratic Republic of Congo or Congo or Cote d'Ivoire or Equatorial Guinea or Eritrea or Eswatini or Ethiopia or Gabon or Gambia or Guinea or Guinea-Bissau or Kenya or Lesotho or Liberia or Madagascar or Malawi or Mali or Mauritania or Mauritius or Mozambique or Namibia or Niger or Nigeria or Rwanda or Sao Tome) and Principe) or Senegal or Seychelles or Sierra Leone or Somalia or South Africa or South Sudan or Sudan or Tanzania or Togo or Uganda or Zambia or Zimbabwe).mp. [mp=title, book title, abstract, original title, name of substance word, subject heading word, floating sub-heading word, keyword heading word, organism supplementary concept word, protocol supplementary concept word, rare disease supplementary concept word, unique identifier, synonyms, population supplementary concept word, anatomy supplementary concept word]	152511
**17.**	15 or 16	**376803**
**18.**	5 and 8 and 14 and 17	**488**
19.	limit 18 to English language	**483**
