# Supplementary material for: Digital health technologies for accessing contraceptive services among young people in Sub-Saharan Africa: A scoping review protocol
Source: PLOS Digit Health. 2025 Jul 10;4(7):e0000748. doi: 10.1371/journal.pdig.0000748 (PMC12244615; doi:10.1371/journal.pdig.0000748)
Supplement: S3 Appendix — (PDF) [file pdig.0000748.s003.pdf]

S3 Appendix: Data Extraction Tool

Scoping review details
Scoping review title:
Review objective:
Review question/s:
Inclusion/exclusion criteria
Population
Concept
Context
Type of source
Details extracted from source of evidence

1. Author/s
2. Title of the source
3. Year of publication
4. Place of publication
5. Country
6. Objective
7. Study methods/design
8. Population
9. Sample size
10. Age
11. Sex/gender
12. Other demographics
13. Settings
14. Intervention or technology characteristics
15. Contraceptive service provided/received
16. Key findings of the paper
17. Key outcome of the intervention
18. Gaps in the intervention
19. Opportunities presented
20. Gaps in evidence
